# Robust Antiferromagnetic FeRh Films on Mica

**DOI:** 10.1021/acsaelm.3c00789

**Published:** 2023-08-31

**Authors:** Alberto Quintana, Carlos Zarco, Nico Dix, Florencio Sánchez, Ignasi Fina, Josep Fontcuberta

**Affiliations:** Institut de Ciència de Materials de Barcelona (ICMAB-CSIC), Campus UAB, Bellaterra 08193, Catalonia, Spain

**Keywords:** flexible electronics, FeRh, anisotropic magnetoresistance, thin films, antiferromagnetic spintronics

## Abstract

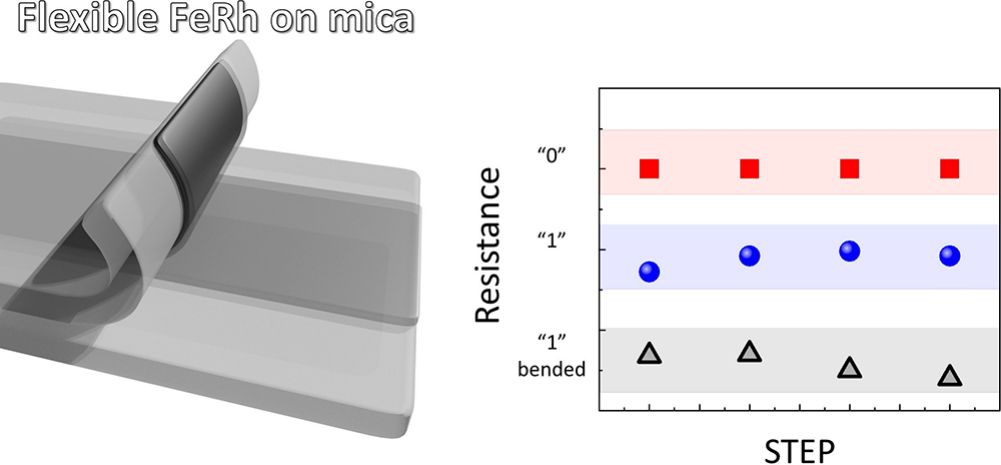

FeRh shows an antiferromagnetic to ferromagnetic phase transition above room temperature, which permits its use as an antiferromagnetic memory element. However, its antiferromagnetic order is sensitive to small variations in crystallinity and composition, challenging its integration into flexible devices. Here, we show that flexible FeRh films of high crystalline quality can be synthesized by using mica as a substrate, followed by a mechanical exfoliation of the mica. The magnetic and transport data indicate that the FeRh films display a sharp antiferromagnetic to ferromagnetic phase transition. Magnetotransport data allow for the observation of two distinguishable resistance states, which are written after a field-cooling procedure. It is shown that the memory states are robust under the application of magnetic fields of up to 10 kOe.

## Introduction

In the last few decades, an increase in the development of flexible functional materials^1−3^ has aimed to respond to the increasing technological demands on wearable and portable devices, prompted by uprising technologies such as the Internet of Things (IoT). Polymeric substrates have been explored for such a goal,^4,5^ demonstrating a high potential as flexible templates for functional films.^6^ However, they impose severe restrictions if high crystalline quality is required. For example, they should be used at relatively low temperatures.^7,8^ Instead, flexible metallic substrates do not have the temperature limit disadvantage and thus allow for the growth of epitaxial films if proper buffer layers are employed.^9,10^

An effective approach to synthesizing highly crystalline/epitaxial flexible films exploits the so-called van der Waals (VdW) heteroepitaxy.^11^ This method takes advantage of weak surface/interface interactions present in 2D and 3D layered materials, due to the lack of dangling bonds at the surface.^12^ Epitaxial growth of thin films has been accomplished using this method even for systems with very high lattice mismatches.^13^ Mica (either muscovite or orphlogopite), while exhibiting strong intralayer bonding, also has weak interlayer interactions, allowing for vdW epitaxy. It is an inexpensive material with very high-temperature stability, conferring similar growth parameters as inorganic substrates but with polymer-like flexibility upon exfoliation,^14,15^ thus appealing for the growth of flexible epitaxial films.^16−18^

Besides, current technology for magnetic data storage is based on ferromagnetic (FM) materials, having a limited data density because of bit-to-bit interactions. FM materials are also sensitive to spurious external magnetic fields, which is detrimental to data storage safety. In contrast, antiferromagnetic (AFM) materials, while having an ordered magnetic structure that allows for data storage, have neighboring moments that are antiparallelly aligned, resulting in an overall zero magnetization. This cancels out bit-to-bit interactions, drastically reduces its sensitivity to external magnetic fields, and enhances data density and safety.^19,20^ Notable progress has been achieved in the development of flexible antiferromagnets, such as Cr_2_O_3_,^21^ IrMn,^22^ Heusler alloys,^23^ or more complex synthetic antiferromagnets.^24^ Typically, these flexible antiferromagnetic materials are envisaged as an instrumental part of spin valve devices.^3,25^ Less explored is the development of flexible FeRh films. Earlier reports demonstrate the synthesis of FeRh over PDMS membranes after a transfer process from sacrificial substrates^26,27^ or direct growth on MgO-buffered metallic tapes.^9^ Any previous report on the use of stand-alone flexible antiferromagnets as memory elements is limited to FeRh on metallic tape.^9^

FeRh alloy, crystallized in the CsCl structure (α′-FeRh), displays a reversible transition from an antiferromagnetic to a ferromagnetic state upon heating.^28,29^ The transition, which occurs at about 75 °C for equiatomic FeRh alloy, can be tuned by either changing the stoichiometric ratio of the alloy or doping with other elements,^30−35^ among other strategies such as hydrostatic pressure,^36^ biaxial strain imposed by the substrate,^37−44^ and local techniques such as ion implantation^45,46^ or indentation.^47^ It was found that by exploiting its antiferromagnetic anisotropic magnetoresistance, FeRh can be used as a memristor.^48^

Previous attempts regarding the development of flexible FeRh thin films faced problems related to the different thermal expansion coefficient between FeRh and the sacrificial layer.^26^ It has been reported that high crystalline quality α′-FeRh films are obtained on MgO vicinal substrates.^49^ In this sense, flexible FeRh films with comparable crystalline quality have already been reported using MgO-buffered flexible metallic tapes.^9^ However, as mentioned, high bending causes the MgO buffer layer to degrade. In addition, metallic tapes show inelastic deformation after repeated bending and the presence of a nonactive metallic element that can act as shunt resistance. As a result, devices may have trouble integrating FeRh films made on buffered metallic tapes. In this work, we report the growth of flexible FeRh films on fluorphlogopite mica substrates and their performance as an antiferromagnetic resistive memory element. The use of mica helps to overcome the drawbacks that metallic tapes present. In addition, we present unprecedented robustness against applications of a large magnetic field of different resistance memory-stabilized states.

## Materials and Methods

### Sample Preparation

**Figure 1:**
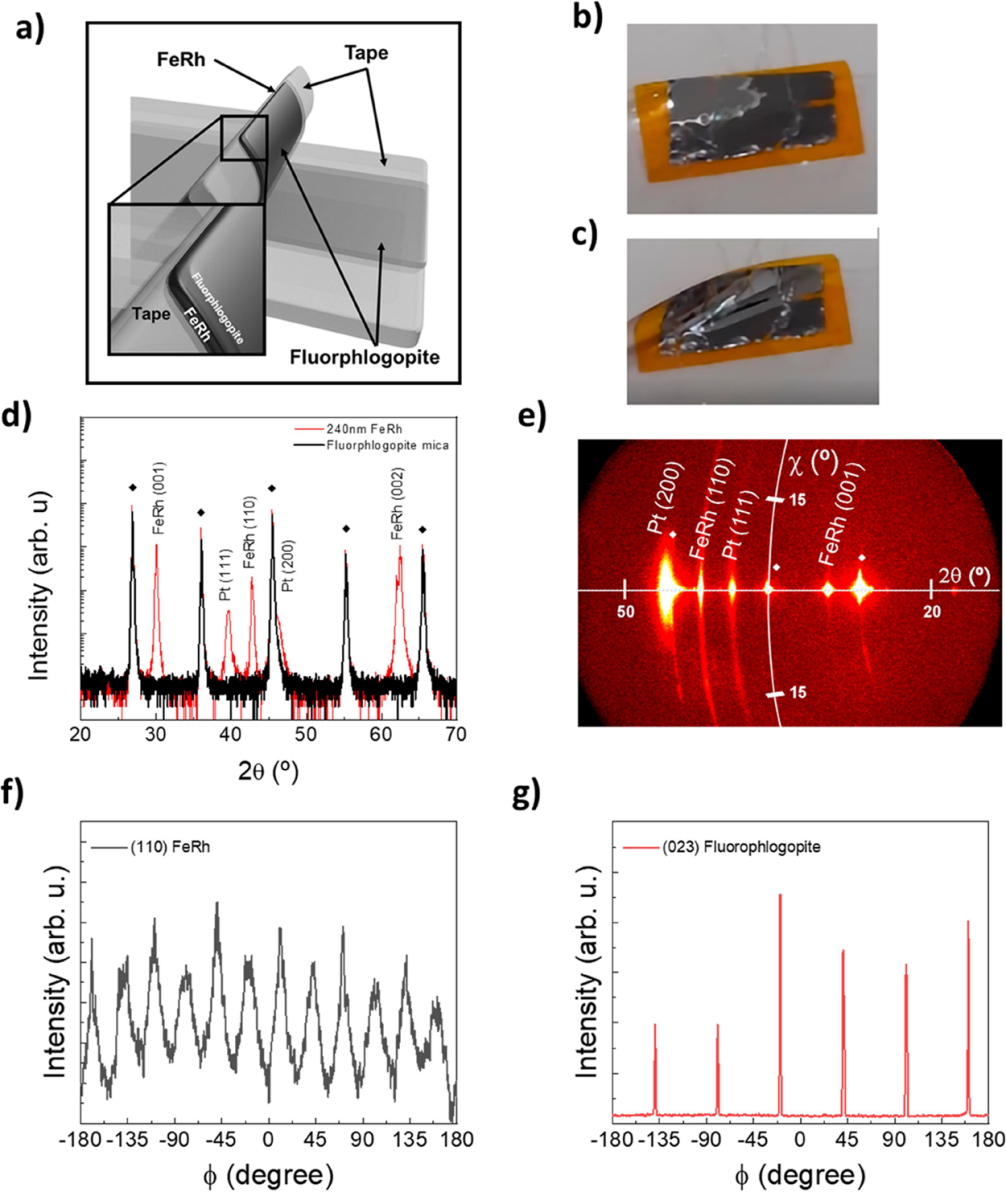
(a) Scheme of the “tape” exfoliation process, (b) photograph of an exfoliated FeRh film (flat) and (c) bended, (d) θ-2θ scans for 240 nm FeRh sample (red) and fluorphlogopite substrate (black), and (e) 2θ–χ scans for 240 nm FeRh film. (f) and (g) φ-scans corresponding to (110) FeRh and (023) fluorphlogopite reflections, respectively. The ◆ symbols in panel (d) account for (00l) reflections of the fluorphlogopite mica substrate.

FeRh films of different thicknesses (60, 120, and 240 nm) were deposited by DC sputtering onto fluorphlogopite substrates from an equiatomic FeRh target. Synthetic fluorphlogopite sheets were purchased from Changchun Taiyuan Co., Ltd., China. A 60 nm FeRh film was also deposited on MgO(001) single-crystal substrates. The deposition temperature (*T*_D_) was 300 °C, and the Ar pressure was 0.01 mbar. Deposited samples were posteriorly annealed in the same chamber at 700 °C and 0.1 mbar Ar during 1 h, as reported elsewhere^50^ and as reported in films grown on single crystalline substrates.^49^ Afterward, samples were cooled to room temperature and subsequently capped with a sputtered Pt (10 nm) protecting layer to avoid FeRh oxidation. Note that at the interface, Pt might result in a change of magnetic anisotropy of FeRh. However, in films thicker than 60 nm with 10 nm Pt, as those characterized here, one should not expect this effect to contribute significantly in the performed functional characterization. Mica was mechanically exfoliated using the “Scotch-tape” method (Figure 1a).^51^Figure 1b,c shows two photographs from an exfoliated FeRh film both flat and bended, respectively.

### Structural Characterization

Structural characterization was performed by X-ray diffraction (XRD) using Cu K_α_ radiation. θ-2θ patterns were acquired using a Siemens D-5000 having a scintillation detector, while 2θ–χ maps and φ-scans were recorded in a Bruker D8 equipped with a 2D detector.

### Magnetic Characterization

Magnetic properties were assessed by using the Quantum Design MPMS-XL Superconducting Quantum Interference Device (SQUID) magnetometer. Electrical characterization was carried out by using a Quantum Design Physical Property Measurement System (PPMS).

### Electrical Characterization

Resistance vs temperature measurements were performed while cooling with a rate of 2 °C/min, in a four-probe collinear configuration covering the whole sample (5 × 5 mm^2^) with an applied current of 1 mA. Anisotropic magnetoresistance (AMR) measurements were performed by initially in-plane field-cooling the sample in a 10 kOe field, applied either parallel (α = 0°) or perpendicular (α = 90°) with respect to the current application direction. Afterward, the external magnetic field was removed, and resistance was measured while warming the sample from 300 to 400 °C. Then, the AMR is calculated as AMR (%) = 100 × (*R*_α=0_ – *R*_α=90_)/*R*_α=90_. Bent configurations were set using 3D-printed holders with an appropriate bending radius (8 mm).

## Results

The X-ray diffraction θ–2θ pattern obtained from a 240 nm thick film is shown in Figure 1d. It shows reflections ascribed to the cubic α′-FeRh (CsCl structure), besides those arising from the mica substrate and the Pt capping layer. No spurious phases are detected. To facilitate the identification of the FeRh diffraction peaks, a θ–2θ scan of the fluorphlogopite substrate has also been included in Figure 1d (black line). FeRh displays a strong (001) out-of-plane texture with a minor presence of (110) textured grains. Note that the intensity of the (110) reflection is about 10 times smaller than the (001). Figure S1 shows θ–2θ patterns for 60 and 120 nm thick samples. The diffractogram of the 240 nm sample has also been included in Figure S1 for ease of comparison. For thinner samples (60 nm), the (110) peak is negligible, while a small increase of (110) intensity is found for 240 nm thickness (Figure S1d). The cell parameters of FeRh extracted from the interplanar distances for (100) and (110) textured phases in the 240 nm thick sample are *a*(100) = 2.990 Å and *a*(110) = 2.991 Å, respectively. Compared to bulk cell parameters of FeRh (*a*bulk = 2.995 Å) confirms that the FeRh 240 nm film is fully relaxed.

2θ–χ scans are taken in the 60, 120, and 240 nm thick FeRh Films. The results are displayed in Figure 1e for 240 nm film and Figure S2 for the other samples. These measurements reveal structural dissimilarities among the two FeRh ([001] and [110]) families of crystallites. Indeed, for all of the samples, the [001] orientation reflections (2θ ≈ 30°) display a small spread along χ, signaling a low mosaicity. On the contrary, the (110) reflections show a thickness-dependent spread along χ, with mosaicity increasing for thicker samples. For comparative purposes, FeRh films were also grown over vicinal MgO (001) substrates using identical growth conditions. MgO has been reported to be an optimal substrate for FeRh growth.^52^Figure S3 displays the structural characterization of the 60 nm FeRh film on the MgO(001) substrate sample and it reveals similar structural properties (mosaicity, intensity, and peak broadening) to those obtained over fluorphlogopite, confirming the high quality of the grown films.

The epitaxial relationship for the grown FeRh films has been assessed by means of φ-scans, and the obtained results are displayed in Figure 1f,g, which correspond to (110) FeRh asymmetric reflections corresponding to the (001) family of crystals and (023) fluorphlogopite reflections, respectively. For the fluorphlogopite (023) peak, due to its monoclinic structure, only two reflections should be observed.^52^ However, fluorphlogopite is prone to have twining defects, resulting in different crystalline stackings, leading to the observed 6 peaks with a 60° separation between them.^53^ Moreover, FeRh (110) is expected to display four reflections due to its cubic structure.^9^ The observed 12 peaks, thus, imply the formation of 3 crystalline domains. These indicate the presence of 3 crystal domains oriented preferentially along [100]FeRh(001) // [010]Mica(001), [100]FeRh(001) // [3-10]Mica(001), and [100]FeRh(001) // [-3-10]Mica(001) (Figure S4).

**Figure 2:**
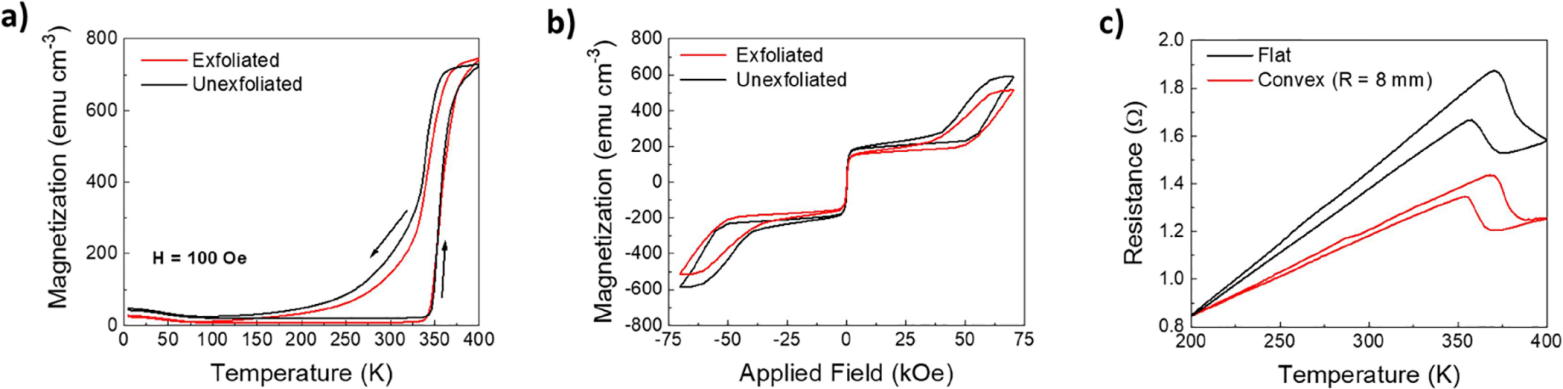
(a) Magnetization vs temperature measurements on a 240 nm FeRh sample in an as-grown (unexfoliated) and exfoliated states, (b) magnetization vs applied field for a nonexfoliated sample and exfoliated sample at 300 K, and (c) resistance vs temperature for a 240 nm exfoliated FeRh sample in flat and bended with a bending radii of 8 mm. Note that data have been vertically shifted for easier comparison.

Figure 2a displays magnetization versus temperature (*M*(*T*)) curves obtained under a 100 Oe in-plane magnetic field for a 240 nm thick film. First, it can be observed that for temperatures close to RT or below, despite the AFM character of FeRh, residual magnetization is observed. This is attributed to the presence of a spurious FeRh FM phase, which may be preferentially located at interfaces.^54^ Upon heating, at about 350 K, a sharp increase in magnetization occurred, as expected for the AFM to FM transition. The observed hysteresis during the heating/cooling process is a fingerprint of the first-order character of the AFM-FM transition.^42^ The hysteretic character can be quantified by defining two critical temperatures (T*_H_ and T*_C_), as the temperature at which the 80% saturation magnetization (*M*_S_) is obtained, at each heating and cooling branch, respectively. Therefore, ΔT* = T*_H_ – T*_C_ quantifies the broadness of the transition. *M*(*T*) measurements have also been acquired for 60 and 120 nm thick samples over fluorphlogopite mica and 60 nm-thick sample over MgO, and results are displayed in Figure S5. *M*(*T*) for the 240 nm sample is given in Figure S5 for the ease of comparison. It can be observed (Figure S5d) that, for increasing thicknesses, ΔT* decreases; thus, the AFM-to-FM transition becomes sharper. Indeed, ΔT* for the 240 nm sample is smaller than for the 60 nm FeRh film grown over MgO (Figure S5d). The sharpness of the transition and the magnetization values at high temperatures confirm the high quality of the synthesized films.^42^

The magnetization data of the FeRh films obtained after exfoliation from the mica substrate are also included in Figure 2a. It can be appreciated that upon exfoliation, T*_H_ slightly decreases, while T*_C_, shifts toward higher temperatures (i.e., narrower hysteresis). The spurious FM phase observed at a low temperature naturally persists, although slightly reduced, after exfoliation. This is consistently observed in all exfoliated samples (Figure S5a–c), implying that exfoliation may enable the films to relax. However, this effect is less pronounced in the 60 nm film, where a larger relaxation is expected, suggesting that the fact that the FM phase ratio is greater also influences the stabilization of the AFM phase.

Hysteresis loops (*M*(*H*)) were also acquired at 300 K and are displayed in Figure 2b. Initially, the sample was saturated with 70 kOe, and an *M*_S_ value of about 600 emu/cm^3^ was recorded. This indicates that the sample has transited from the AFM into the ferromagnetic state upon magnetic field application.^49^ The difference in *M*_S_ values between *M*(*H*) and *M*(*T*) measurements indicates that a small AFM fraction still has not transited into the FM state, decreasing the saturation magnetization. Scanning down the applied magnetic field, at about 40 kOe, a sharp decrease in magnetization is observed, which is due to the FM phase transiting back into the AFM one. As observed for the temperature dependence, the magnetic field-induced transition is also hysteretic. The magnetic field at which the AFM-to-FM transition occurs is also lowered after substrate exfoliation. At low fields (<1 kOe), the hysteretic behavior is attributed to the residual ferromagnetic phase.

Resistance has been measured in a 240 nm exfoliated sample, either in flat shape or bent (bending radii of 8 mm), during zero-field cooling from 400 to 200 K and during the subsequent warming up. Note that, upon bending, the in-plane tensile strain of 0.2% is produced (Figure S6). The obtained data have been shifted to their value at 200 K for ease of comparison and are displayed in Figure 2c. Data reveal that resistance increases with the increasing temperature, as expected for the metallic character of FeRh. A drop in the resistance is observed after crossing the transition temperature due to the lower resistance of the FM state when compared to the AFM phase.^50^ Similar measurements were taken upon bending with radii of 8 mm (red line in Figure 2c). *R*(*T*) results for the bent configuration show that the slope of the *R*(*T*) is reduced. Note also that the resistance drop across the AFM-to-FM transition is larger for the flat configuration. Considering that, if in the probed region FM domains completely transform to AFM ones while cooling, a large resistance change would be observed, whereas if no transformation is observed, no resistance change would be observed; the larger resistance drop in the flat configuration indicates a larger AFM fraction compared with the bent configuration.

**Figure 3:**
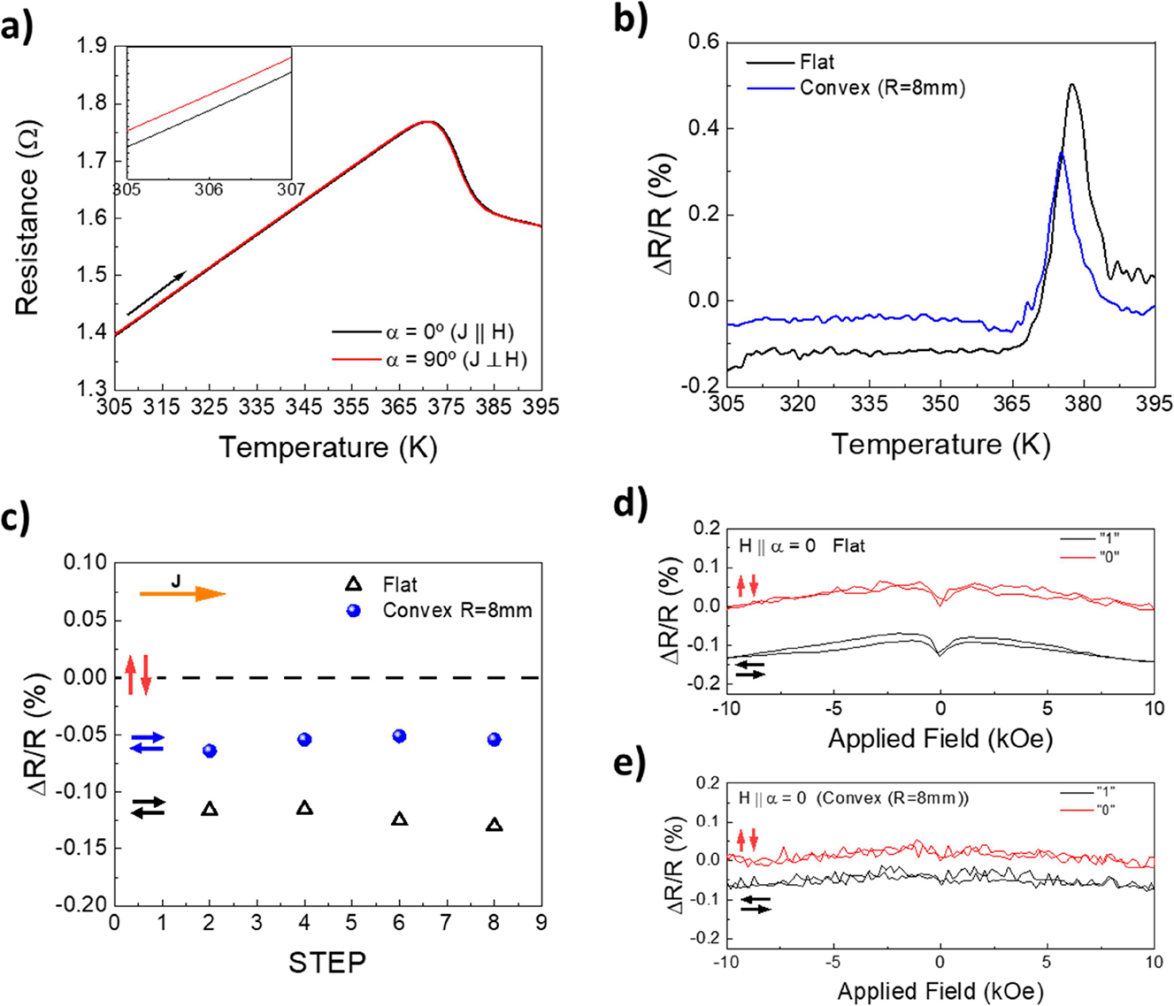
(a) Zero-field heating resistance vs temperature curves for samples previously field-cooled along the measurement axis (α = 0°) and perpendicular to it (α = 90°). Inset displays zoom-in view for the 305–307 K range showing different resistance for α = 0° and α = 90°. In (b), Δ*R*/*R* (%) vs temperature, where Δ*R* stands for *R*_α=0_ – *R*_α=90_ and *R* = *R*_α=90_. (c) memory (Δ*R*/*R*) cyclability for a 240 nm FeRh film in flat and bended (convex) with a bending radii of 8 mm. (d) Flat and (e) convexly bended, Δ*R*/*R* vs applied filed (i.e., memory robustness measurements) for the different memory states with the magnetic field applied along α = 0.

Finally, the possibility of employing the synthesized and exfoliated FeRh films as flexible memory elements has been assessed by exploring its anisotropic magnetoresistance (AMR) properties.^50^ Two different resistance states (memory states “1” and “0”) have been set field-cooling under the 10 kOe sample from the highest temperature (395 K) to the measuring temperature, along two in-plane orthogonal directions. Note that the current is always injected in the same direction. Therefore, α = 0° stands for the field-cooling direction with the magnetic field parallel to the current injection direction (memory state “1”), and α = 90° for the orthogonal one (memory state “0”). After the field-cooling procedure, the magnetic field was switched off and the resistances *R*_α=0_ and *R*_α=90_ were recorded while heating from 305 up to 395 K. Figure 3a displays the *R*_α=0_ and *R*_α=90_ curves obtained in a 240 nm thick unbent sample. A well-perceptible change of resistance is observed (see the inset of Figure 3a). The AMR is calculated using the expression AMR (%) = 100 × (*R*_α=0_ – *R*_α=90_)/*R*_α=90,_ and it is plotted as a function of the temperature in Figure 3b. A resistance change (Δ*R*/*R*) of −0.12% is obtained at room temperature (305 K). This value is much larger than that obtained in FeRh films on metallic tapes,^9^ probably due to the presence of the metallic tape that acted as a shunt resistance. Upon heating, the AMR value is preserved up to 365 K, where the AFM-to-FM transition is triggered. During the transition, a drastic increase in Δ*R*/*R* is observed, reaching a maximum value of +0.5%, which is a consequence of the different resistance states depending on the previous field-cooling direction, which impacts the T*_H_ position.^55^Figure 3b also shows the AMR(*T*) values recorded on the bent FeRh film (convex, bending radii of 8 mm). Data for the bent film clearly indicate that AMR persists (AMR ≠ 0) and thus, the magnetically written information is preserved. The AMR magnitude is slightly reduced (−0.06%) compared with the unbent sample (−0.12%). This last observation can be explained by recalling that tensile strain is generated in the film during convex bending, which promotes the transition into the FM phase, as previously reported.^56^ In Figure 3c, we show AMR values at 305 K both in the flat sample and in the bent state, one sequentially recorded after repeated sample cooling while applying a magnetic field along parallel and perpendicular directions. It is clear that measurements and states are reproducible and there is little dispersion in Δ*R*/*R* values. Note the small drift in the Δ*R*/*R*, whose origin is difficult to disclose with the available data. Finally, the memory robustness is probed setting a memory state and subjecting the sample to in-plane magnetic fields up to 10 kOe along two orthogonal directions for every memory state. Figure 3d,e show resistance vs magnetic field curves, carried out at room temperature, for the 240 nm FeRh sample in both flat and bent configurations, respectively. For these measurements, an external magnetic field was applied along α = 0°, for both memory states “1” and “0”. It can be observed that the initial Δ*R*/*R* value remains unaltered, after the application of a magnetic field as large as ±10 kOe, even with the mentioned presence of residual FM phase as shown in Figure 3b. Similar results have been obtained in the bent state (Figure 3e), despite the somewhat larger noise level in this measurement due to experimental shielding limitations associated with the used holder for sample bending. Note that the slope at high field results most probably from, ordinary, Lorentz force magnetoresistance that is independent of spin. In the not-bend experiment, at a near-zero magnetic field (Figure 3d), there is an inverted peak in the resistance resulting from residual ferromagnetic contributions. This is not visible in the bent state (Figure 3e), as a result of the mentioned larger noise level. Note that under a magnetic field, the resistance state varies due to the mentioned presence of ordinary MR. This implies that under a magnetic field, two different resistance states might not be distinguishable. However, after the removal of the large magnetic field, that is, at remanence, the initial resistance states are recovered, and the contrast between “1” and “0” states can be well appreciated.

Figure S7 shows equivalent data for magnetic fields applied along α = 90°. This result shows that different magnetic textures corresponding to different resistance states, which are robust under the application of a large magnetic field, can be stabilized. For applications, thermal heating might not be sufficiently efficient. Therefore, the antiferromagnetic state should be manipulated in a more efficient manner, that is, using current-induced fields^57^ or spin–orbit-torque,^58^ or using Joule heating induced by current as a local heating source.^59^

## Conclusions

In summary, we have demonstrated the possibility of growing epitaxial (001) - FeRh thin films over flexible fluorphlogopite mica substrates. In spite of the presence of two families of epitaxial crystallites, (001)- and (110)-oriented, sharp and narrow AFM-to-FM transition is observed in the thin films, with magnetizations of about 750 emu/cm^3^ at 400 K, comparable to those of FeRh epitaxial films grown on MgO single crystal substrates. Flexibility (bending radii up to 8 mm) on the deposited films is improved upon mica exfoliation. Its performance as a flexible AFM memory resistor is demonstrated in a repeatable manner (up to 8 times), obtaining AMR values of about 0.1% without degradation using bending radii up to 8 mm. The identification of AMR in flexible films represents a significant step forward in the creation of flexible resistance memory based solely on antiferromagnetic materials. To achieve this objective, a more systematic characterization of the mechanical loading properties of the material is required.

## Supporting Information Available

Extended structural and magnetic characterization; diagram of the bending geometry; and strain determination (PDF)
