# Supplementary material for: Robust Antiferromagnetic FeRh Films on Mica
Source: ACS Appl Electron Mater. 2023 Aug 31;5(9):5043–9. doi: 10.1021/acsaelm.3c00789 (PMC10540149; doi:10.1021/acsaelm.3c00789)
Supplement: Supplementary file 1 — el3c00789_si_001.pdf [file el3c00789_si_001.pdf]

Supporting Information

Robust antiferromagnetic FeRh films on mica

Alberto Quintana, Carlos Zarco, Nico Dix, Florencio Sánchez, Ignasi Fina*, Josep Fontcuberta*

Institut de Ciència de Materials de Barcelona (ICMAB-CSIC), Campus UAB, Bellaterra 08193, Catalonia, Spain

**Corresponding authors: ifina@icmab.es (Ignasi Fina); fontcuberta@icmab.cat (Josep Fontcuberta)*

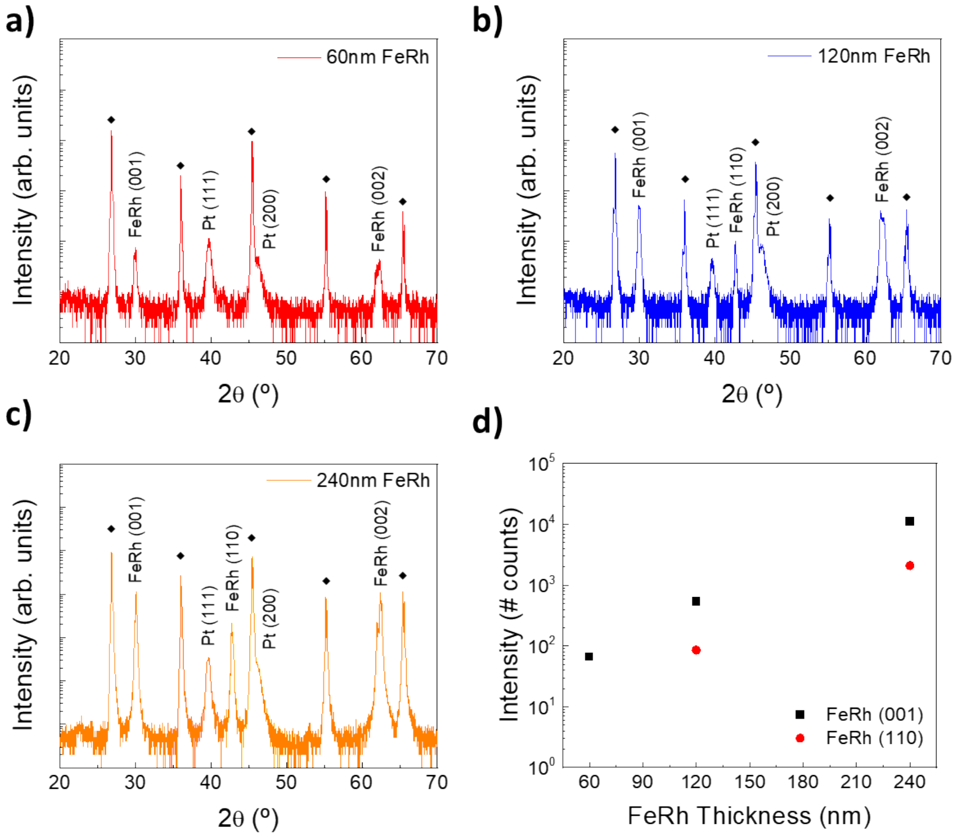

Fig. S1: θ-2θ scans for FeRh films on Fluorphlogopite substrate **a)** 60nm and **b)** 120nm. In **c)** 240nm thick has been included for ease of comparison. **d)** FeRh XRD peak intensity for different FeRh thicknesses and crystallographic reflections ((001) and (110)).

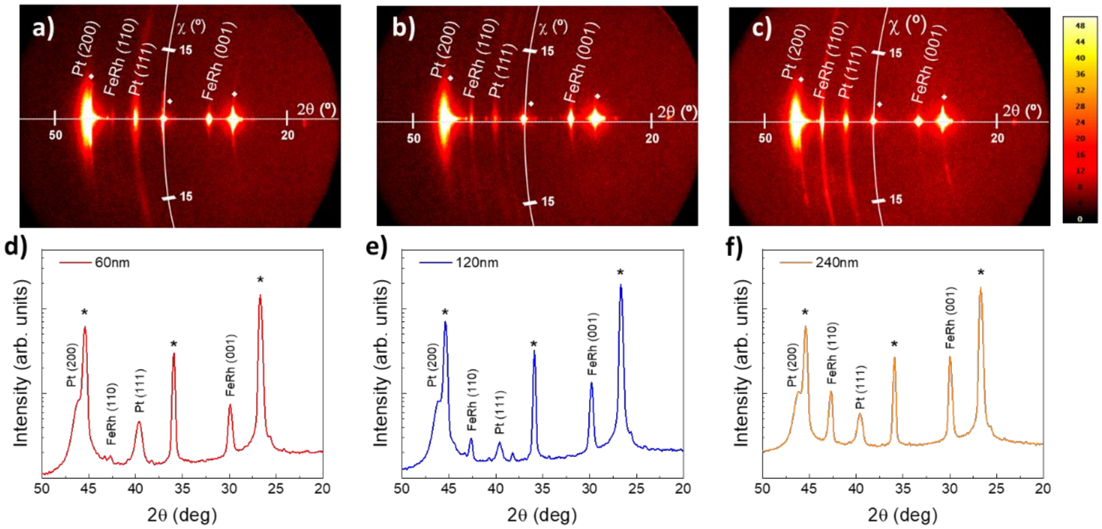

Fig. S2: 2θ – χ scans for FeRh films on Fluorphlogopite substrate **a)** 60nm and **b)** 120nm. In **c)** 240nm thick has been included for ease of comparison. In **d), e)** and **f)** Intensity vs 2θ plots obtained from 2θ-χ integration along χ-axis. Note the inverted 2θ axis in d)-f) .

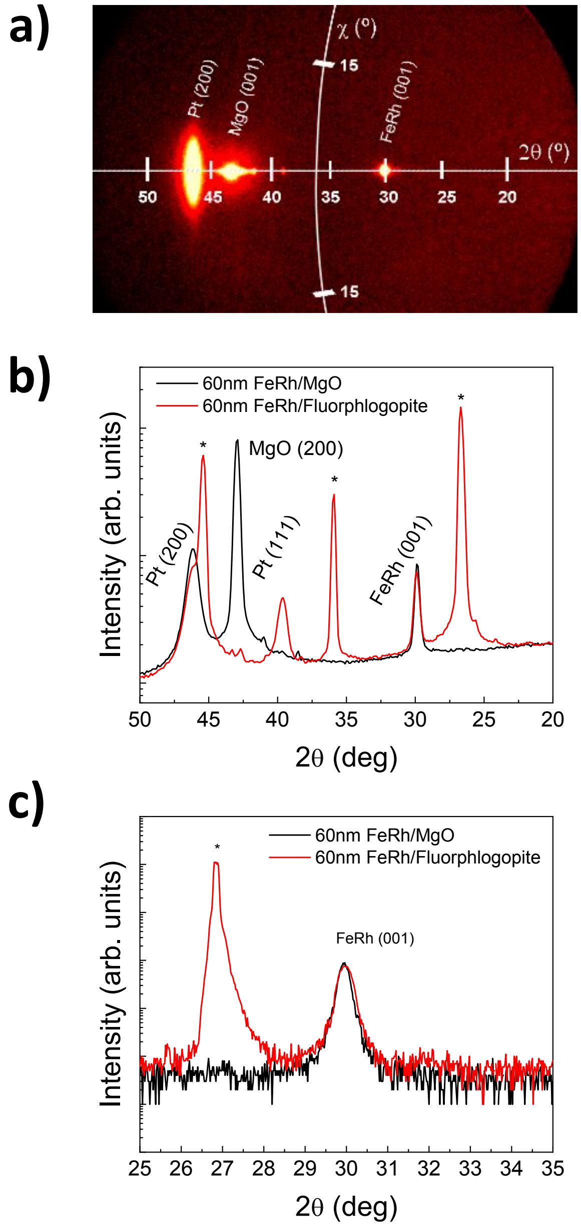

Fig. S3: a) 2θ – χ scans for FeRh films on MgO substrate. b) 2θ scans for the 60nm FeRh films on MgO and Fluorphlogopite mica obtained integrating the 2θ – χ scans. In c) zoom in view of the 25-35º region from 2θ pattern in b).

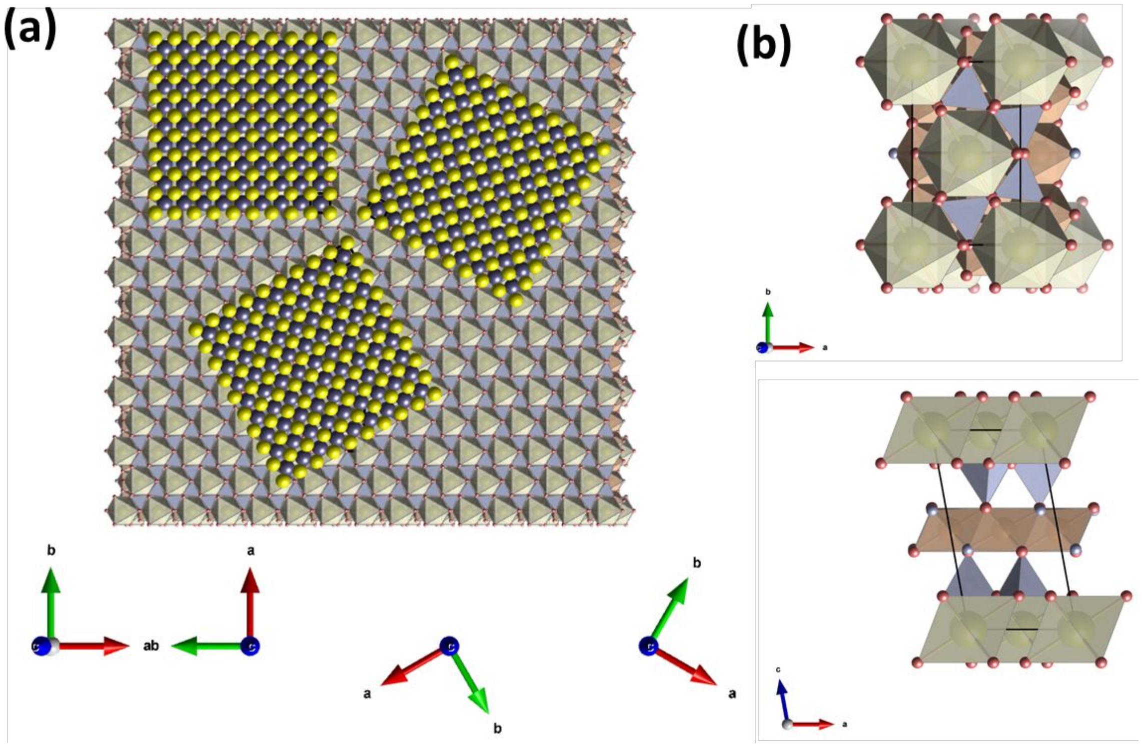

Fig. S4: a) Scheme of the three different in-plane growth orientations of FeRh over fluorphlogopite mica substrate. **b)** Scheme of the out-of-plane growth of FeRh over fluorphlogopite mica.

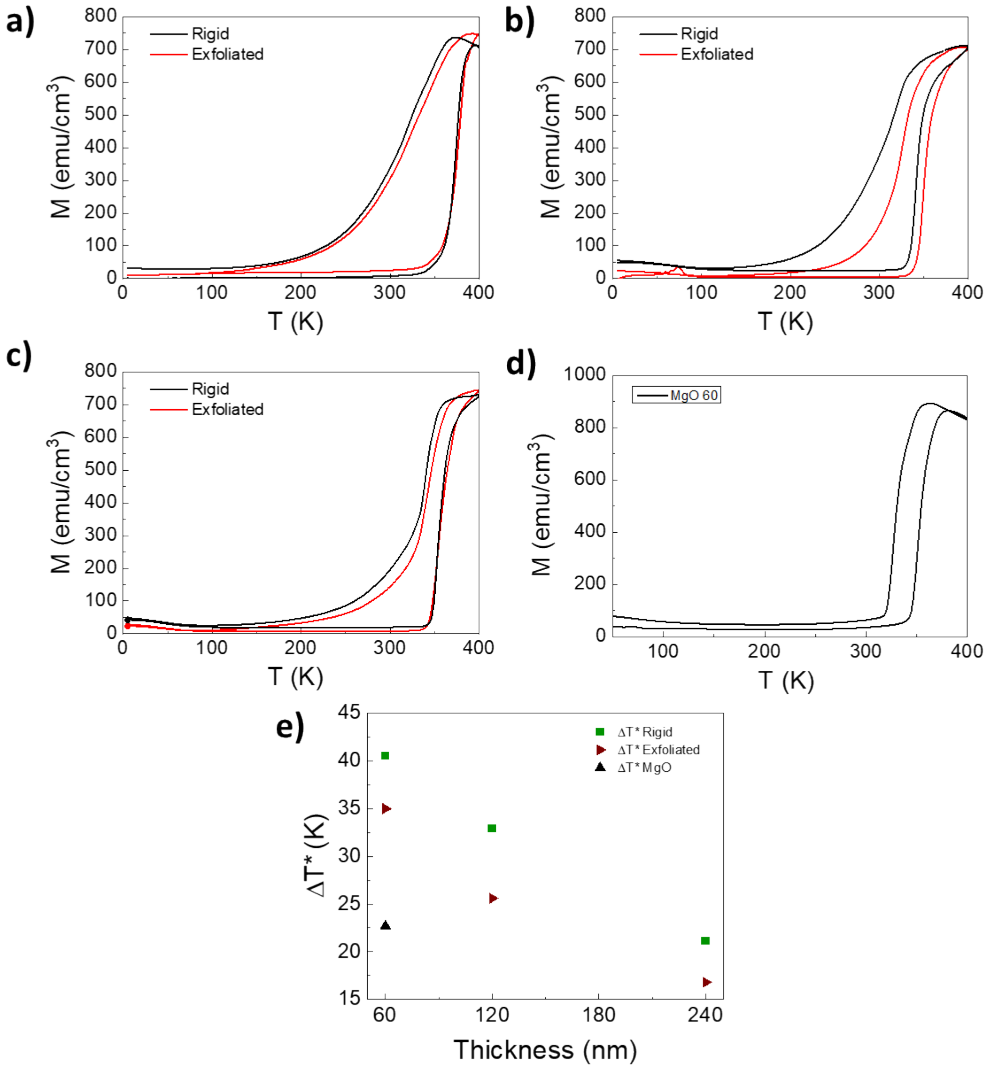

Fig. S5: a) magnetization vs temperature measurements before and after exfoliation of the Fluorphlogopite substrate for **a)** 60nm **b)** 120nm and **c)** 240nm thick FeRh films. In **d)** the magnetization vs temperature curve for the FeRh/MgO sample. **e)** ΔT* as a function of FeRh film thickness (either as-grown and exfoliated) and MgO single crystal sample.

Calculation of the produced strains upon bending

Strain, ε, is defined as (equation 1):

$$\varepsilon = \frac{\Delta L}{L_0} \qquad (1)$$

Where ΔL is the change in size after deformation and L_0 is the initial size of the sample. From the scheme shown in Figure 1, it is possible to rewrite equation 1 in terms of the sample thickness, substrate thickness and the bending radii (equation 2).

$$\varepsilon = \frac{\Delta L}{L_0} = \frac{L_f - L_0}{L_0} = \frac{\left(\left(t + \frac{Y}{2}\right) + R\right)\theta - R\theta}{R\theta} = \frac{t + {}^{Y}/_{2}}{R} \qquad (2)$$

Where L_f stands for the final length of the sample upon bending, L_0 is the initial length, t is the FeRh film thickness, Y is the remaining mica thickness, R and θ are, respectively, the bending radii and bending angle.

Since the remaining mica thickness (~30micron) is much larger than the FeRh thickness (240nm), equation 2 can be simplified as:

$$\varepsilon = \frac{Y}{2R} \qquad (3)$$

Thus, for a bending radii of 8mm and sample configuration, the generated strain is about 0.2%.

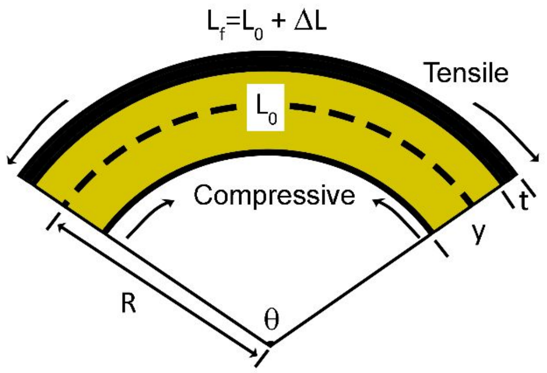

Fig. S6: Scheme of the convex bending. In dark, with thickness t, the FeRh Film and in yellow with thickness Y, the remaining mica after exfoliation.

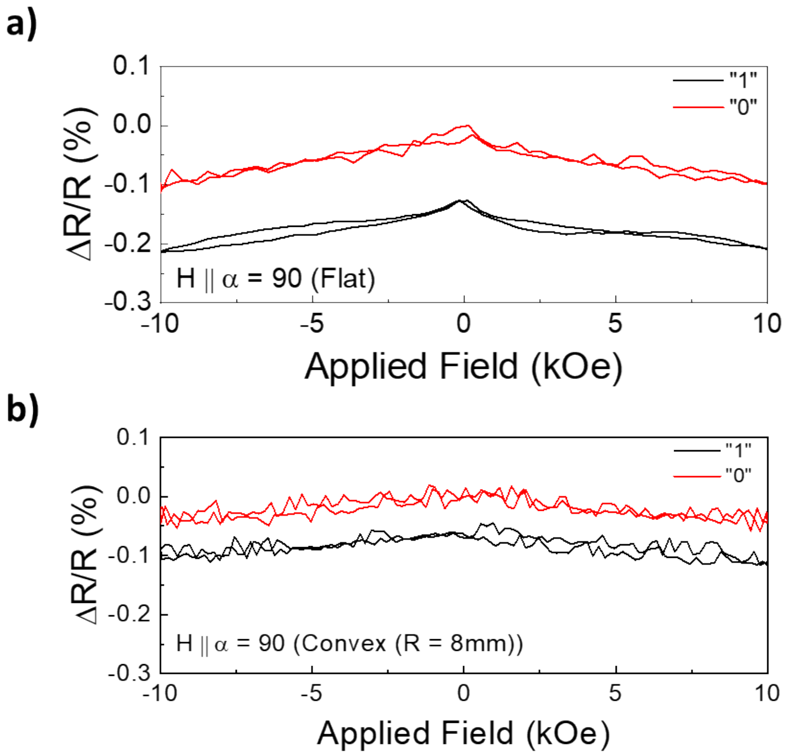

Fig. S7: $\Delta R/R$ vs applied filed (i.e. memory robustness measurements) for the two memory states with the magnetic field applied along α=90° for **(a)** flat and **(b)** convex (R = 8mm) configuration.
